# Association between coffee consumption and risk of recurrent venous thromboembolism – Results from the Trøndelag Health Study and Tromsø Study

**DOI:** 10.1016/j.rpth.2026.106966

**Published:** 2026-09-15

**Authors:** Melliane M. Olsen, Sigrid K. Brækkan, Kristian Hveem, Kjersti Grønning, John-Bjarne Hansen

**Affiliations:** 1 Nord-Trøndelag Hospital Trust, Norway; 2 Thrombosis Research Group (TREC), Department of Clinical Medicine, UiT – The Arctic University of Norway, Tromsø, Norway; 3 Thrombosis Research Center (TREC), Division of Internal Medicine, University Hospital of North Norway, Tromsø, Norway; 4 HUNT Center for Molecular and Clinical Epidemiology, Department of Public Health and Nursing, Faculty of Medicine and Health Science, Norwegian University of Science and Technology, Trondheim, Norway; 5 Department of Public Health and Nursing, HUNT Research Center, Norwegian University of Science and Technology, Levanger, Norway; 6 Department of Research, St. Olav University Hospital, Trondheim, Norway; 7 Department of Public Health and Nursing, Norwegian University of Science and Technology, Trondheim, Norway

**Keywords:** coffee, pulmonary embolism, recurrence risk, venous thromboembolism, venous thrombosis

## Abstract

**Background:**

Epidemiological studies have shown that coffee consumption is associated with a lowered risk of incident venous thromboembolism (VTE). No previous study has explored whether coffee consumption influences the risk of VTE recurrence.

**Objectives:**

This study aimed to investigate whether the amount of coffee consumption (cups/d) is associated with the risk of VTE recurrence in a population-based cohort of incident VTEs.

**Methods:**

The cohort consisted of 2071 patients with incident VTE recruited from 4 surveys of the Tromsø Study (enrolment, 1994-2008) and the third survey of the Trøndelag Health Study (enrolment, 2006-2008), followed from the date of the index VTE until 2020. Information on coffee consumption (cups/d) and potential confounders was extracted. Cox regression models were used to estimate hazard ratios (HRs) with 95% CIs for VTE recurrence.

**Results:**

During a median follow-up of 2.8 years, 310 recurrent VTEs occurred. There was no linear relationship between increasing coffee intake and VTE recurrence. The HR for recurrence was 0.49 (95% CI, 0.30-0.81) for those consuming 1 to 2 cups of coffee per day and 0.71 (95% CI, 0.44-1.12) for those consuming >6 cups compared with noncoffee consumers. The association between coffee consumption (yes/no) and lowered recurrence risk was more pronounced for provoked index VTE event (HR, 0.50; 95% CI, 0.30-0.82) than for unprovoked index VTE (HR, 1.01; 95% CI, 0.53-1.94).

**Conclusion:**

Coffee consumption was associated with a nonlinear, lowered risk of VTE recurrence, particularly after provoked VTE. Our findings suggest that coffee can be safely consumed after VTE, potentially with a beneficial effect on recurrence risk.

## Essentials

• Whether coffee consumption influences the risk of venous thromboembolism (VTE) recurrence has not been explored.
• We investigated the association between coffee and VTE recurrence in 2071 patients with VTE.
• Coffee consumption was associated with a 30% to 50% lower risk of VTE recurrence.
• Coffee can be safely consumed after VTE, potentially with a beneficial effect on recurrence risk.

## 1 Introduction

Venous thromboembolism (VTE), encompassing deep vein thrombosis (DVT) and pulmonary embolism (PE), affects 1 in 12 individuals during their lifetime [1] and over 10 million individuals worldwide annually [2,3], frequently followed by severe complications and mortality [4,5]. Among survivors of the incident VTE event, the individual suffering and burden on health care systems and the societal economy [6,7] are high due to long-term complications such as postthrombotic syndrome [8,9], post-PE syndrome [10], recurrence [5,11], poor quality of life [12,13], depression [14], and increased likelihood of being granted disability pension [15]. Approximately 30% of patients with VTE experience a recurrent event within 10 years after their incident event, of which most occur within the first years [5,11]. The risk of severe complications and death is higher after VTE recurrence compared with the incident event [[16], [17], [18], [19]]. Extended anticoagulant treatment can efficiently reduce the risk of VTE recurrence, but at the expense of increased risk of major bleeding [20,21]. Therefore, it is important to identify lifestyle factors associated with lowered risk of VTE recurrence.

Coffee is one of the most widely consumed alcohol-free beverages in the world [22,23]. Several epidemiological studies have reported that coffee consumption is associated with a 15% to 33% lowered risk of incident VTE [[24], [25], [26], [27]] and that the inverse association between coffee consumption and PE is stronger than for DVT [27]. Coffee and constituents of coffee are known to affect several pathways implicated in the pathogenesis of VTE [26,[28], [29], [30], [31]], supporting a biological link between coffee consumption and VTE risk.

No previous study has, to the best of our knowledge, investigated the association between coffee consumption and the risk of VTE recurrence. We hypothesized that coffee consumption was associated with a lowered risk of VTE recurrence. Using data on incident VTEs and self-reported coffee consumption registered prior to the incident VTE derived from 2 large population-based cohorts, we investigated whether coffee consumption was associated with the risk of VTE recurrence.

## 2 Methods

### 2.1 Study population

The Tromsø Study and the Trøndelag Health Study (HUNT) are Norwegian population-based cohorts comprising inhabitants of the Tromsø Municipality and the former Nord-Trøndelag County, respectively. A total of 36,626 women and men aged 25 to 99 years attended the Tromsø 4 to 7 surveys (enrolment in 1994-2016) [32,33], while 50,807 women and men aged 20 to 101 years attended the HUNT3 survey (enrolment in 2006-2008) [34,35]. These 2 surveys were combined to form a large cohort, and all VTE events occurring in the follow-up period (up to 2019-2020) were thoroughly identified, validated, and recorded as previously described in detail [36,37]. In total, 2071 individuals who were diagnosed with symptomatic, objectively confirmed first-lifetime VTE comprised our study population (Figure 1).

**Figure 1:**
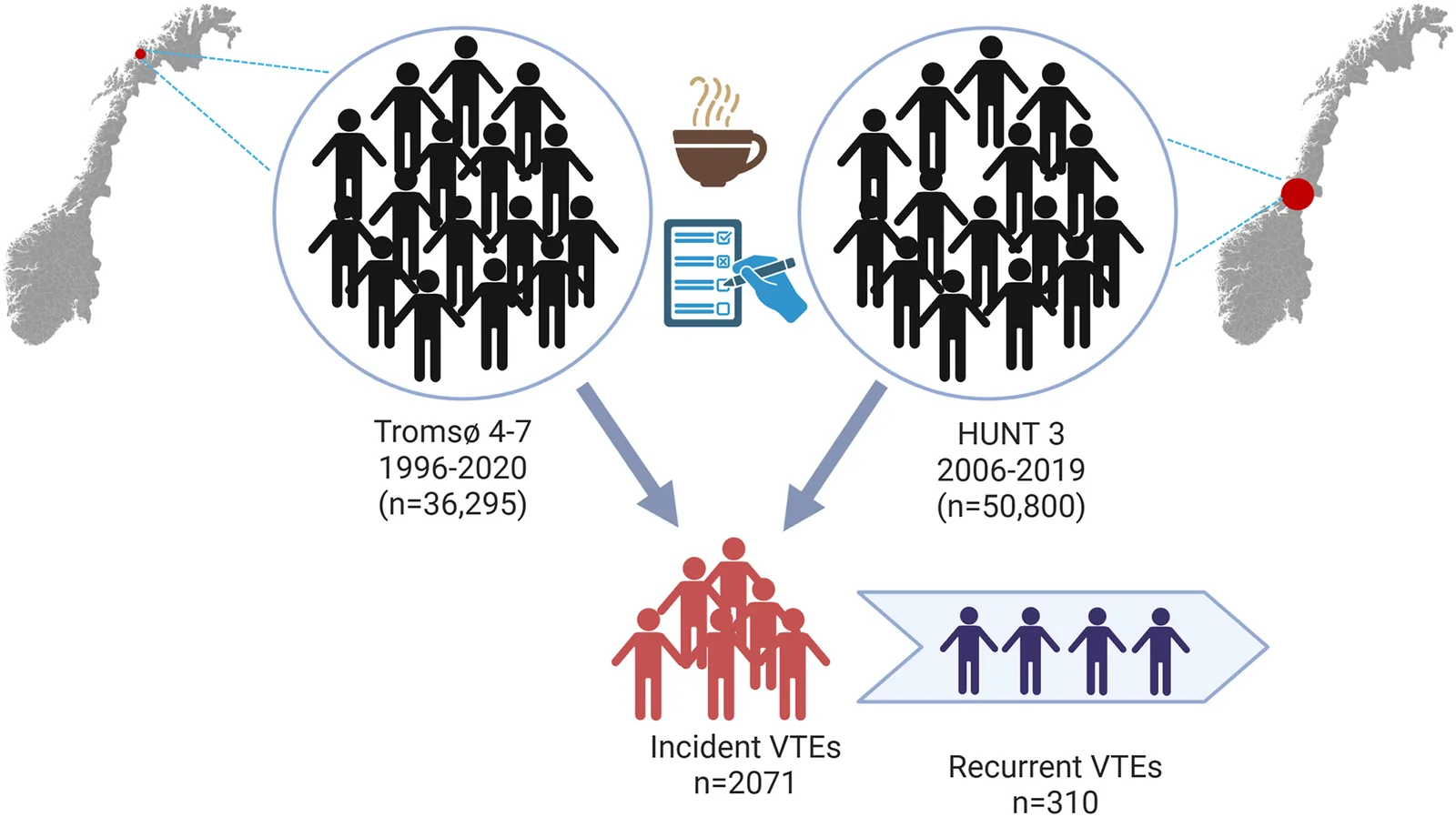
Study population.

All participants gave their informed and written consent to participate in Tromsø and HUNT and gave permission for use of their collected data in research. The study was approved by the Regional Committee for Health and Research Ethics in North Norway (REK nr 42460).

### 2.2 Baseline measurements

Information about the participants was collected in each survey by questionnaires (self-report), physical examination, and blood samples. For those who had attended multiple cohort surveys (Tromsø), we used the information from the survey closest preceding the index VTE. The physical examination included measurements of body height and weight, with participants dressed in light attire and with no shoes. Body mass index (BMI) was calculated in kg/m^2^. Information on prevalent cardiovascular diseases (CVDs; myocardial infarction, stroke, and angina) and cancer, physical activity, smoking habits, and coffee consumption was collected through the questionnaires. The questions on coffee intake were as follows: “How many cups of coffee do you usually drink daily?” and the participants were asked to fill in the number of cups of “boiled coffee” (coarsely ground coffee for brewing) or “other types of coffee” consumed per day. Based on this information, we generated a variable for total daily coffee consumption and categorized it as follows: “no-coffee consumption,” “1 to 2 cups/d,” “3 to 4 cups/d,” “5 to 6 cups/d,” and “>6 cups/d.”

### 2.3 VTE recurrence identification and validation after the first VTE

The first-time VTEs (*n* = 2071) and recurrent events among these occurring within the study period (1994-2020) were identified by searching through the hospital registries at the University Hospital of North Norway (Tromsø), and the 2 local hospitals Levanger and Namsos, as well as the regional tertiary-care center, St. Olavs Hospital in Trondheim, as previously described [36,37]. Trained health care professionals reviewed the medical record for each identified VTE event. These VTE events were validated and recorded when signs and symptoms of DVT or PE were present, followed by objective confirmation of a thrombus by radiological imaging or autopsy, and anticoagulant treatment was initiated, unless contraindicated [37]. When concomitant PE and DVT were diagnosed, the event was classified as PE. Information on provoking factors preceding each event was recorded using a standardized form. A VTE was categorized as either unprovoked or provoked, depending on the presence of more than one provoking factor. Provoking factors included recent surgery or trauma within the past 8 to 12 weeks, acute medical conditions (such as acute myocardial infarction, ischemic stroke, or a major infectious disease), active cancer, significant immobilization (defined as having bed rest for >3 days or wheelchair use within 8 weeks prior to the event, or long-distance travel exceeding 4 hours within the 2 weeks prior to the event), or other provoking factor(s) specifically documented by a physician in the medical record (eg, an intravascular catheter).

Identification and validation of VTE recurrences followed the same procedure as the first VTE events and required new onset of signs and symptoms of VTE, followed by objective confirmation using imaging (or autopsy).

### 2.4 Statistical analysis

Stata version 18.5 (StataCorp) was used to perform statistical analyses. All participants (*n* = 2071) were followed from the date of their first-lifetime VTE until the occurrence of a recurrent VTE, migration, death, or the end of the follow-up period. The crude incidence rates (IRs) of recurrence were estimated by dividing the number of VTE recurrences by the total person-years at risk within each coffee consumption category and expressed as events per 100 person-years. Cox proportional hazards regression models were used to estimate hazard ratios (HRs) with 95% Cls for VTE recurrence according to increasing coffee consumption categories (0 cups/d was used as the reference category) and according to coffee consumption (yes/no). The analyses were adjusted for age and sex (model 1) and for age, biological sex, BMI, history of CVD, and cancer (model 2). We additionally performed subgroup analyses according to the type of index event (PE, DVT, unprovoked and provoked VTE). The cumulative incidences of recurrence according to coffee consumption categories were displayed using 1-Kaplan-Meier curves.

We did not have exact information on the duration of anticoagulant treatment. We therefore conducted sensitivity analyses in which the index date (ie, start of follow-up) was set to 3 months and 6 months after the first VTE, as this duration would represent the period(s) when the initial anticoagulation was completed in most cases.

## 3 Results

The population characteristics and clinical presentation of the incident VTE events across categories of coffee consumption are shown in Table 1. Participants who reported no-coffee consumption at the time of recurrence VTE diagnosis were younger (mean age 60 years) than those reporting 1 to 2 cups/d of regular coffee consumption (mean age 73 years), whereas the proportions of men and smokers increased with categories of increasing coffee consumption. The proportions of DVT as the index event increased, whereas PE decreased over categories of increasing amounts of coffee consumption. Of note, the proportions of DVTs, PEs, and unprovoked VTEs were essentially similar in patients who did not drink coffee and those who drank 1 to 2 cups of coffee daily.

**Table 1:** Baseline characteristics of the Trøndelag Health Study (HUNT) and Tromsø Study (1994-2020) population across categories of coffee consumption.

Characteristics	No-coffee consumption	1-2 cups/d	3-4 cups/d	5-6 cups/d	>6 cups/d
n	140	368	698	534	331
Age (ys)	60 ± 16	73 ± 13	73 ± 12	69 ± 12	64 ± 12
Sex (female, %, n)	53 (74)	59 (218)	52 (364)	47 (251)	41 (136)
BMI (kg/m^-2^)	28.2 ± 5.7	28.3 ± 4.9	27.7 ± 4.1	28.1 ± 4.8	27.7 ± 4.9
Smoking (%, n)	16.4 (23)	7.1 (26)	13.2 (92)	26.0 (139)	46.2 (153)
CVD (%, n)	11.4 (16)	17.4 (64)	15.0 (105)	10.3 (55)	10.6 (35)
Characteristics of index VTE, % (n)					
Deep vein thrombosis	46.4 (65)	48.1 (177)	50.1 (350)	49.6 (265)	56.2 (186)
Pulmonary embolism	53.6 (75)	51.9 (191)	49.9 (348)	50.4 (269)	43.8 (145)
Unprovoked VTE	45.7 (64)	47.3 (174)	39.7 (277)	41.4 (221)	42.6 (141)
Provoked VTE	54.3 (76)	52.7 (194)	60.3 (421)	58.6 (313)	57.4 (190)
Active cancer	20.0 (28)	22.3 (82)	24.5 (171)	24.2 (129)	22.7 (75)
Surgery	15.0 (21)	16.6 (61)	17.3 (121)	15.6 (83)	17.5 (58)
Trauma	10.7 (15)	11.7 (43)	12.8 (89)	10.7 (57)	8.8 (29)
Acute medical condition	7.1 (10)	6.0 (22)	8.2 (57)	6.0 (32)	10.0 (33)
Immobility	21.4 (30)	22.3 (82)	22.5 (157)	22.3 (119)	20.2 (67)
Other provoking factors	7.1 (10)	3.5 (13)	3.7 (26)	3.0 (16)	4.5 (15)

*n* = number of observations.

BMI, body mass index; CVD, cardiovascular disease; VTE, venous thromboembolism.

There were 310 recurrent VTEs during a median follow-up of 2.8 years, corresponding to an overall recurrence rate of 3.3 (95% CI, 3.0-3.7) per 100 person-years. The cumulative incidence of overall VTE recurrence across categories of coffee consumption and first VTE events is shown in Figure 2. The cumulative incidences of VTE recurrence were 15% at 1 year, 32% at 3 years, and 45% at 5 years among no-coffee consumers. The corresponding cumulative incidences were 8%, 20%, and 30% for those who reported a consumption of 1 to 2 cups of coffee per day, while the cumulative incidence curves for those who consumed 3 to 4 cups/d, 5 to 6 cups/d, and >6 cups/d fell between the curves for the coffee abstainers and those drinking 1 to 2 cups/d.

**Figure 2:**
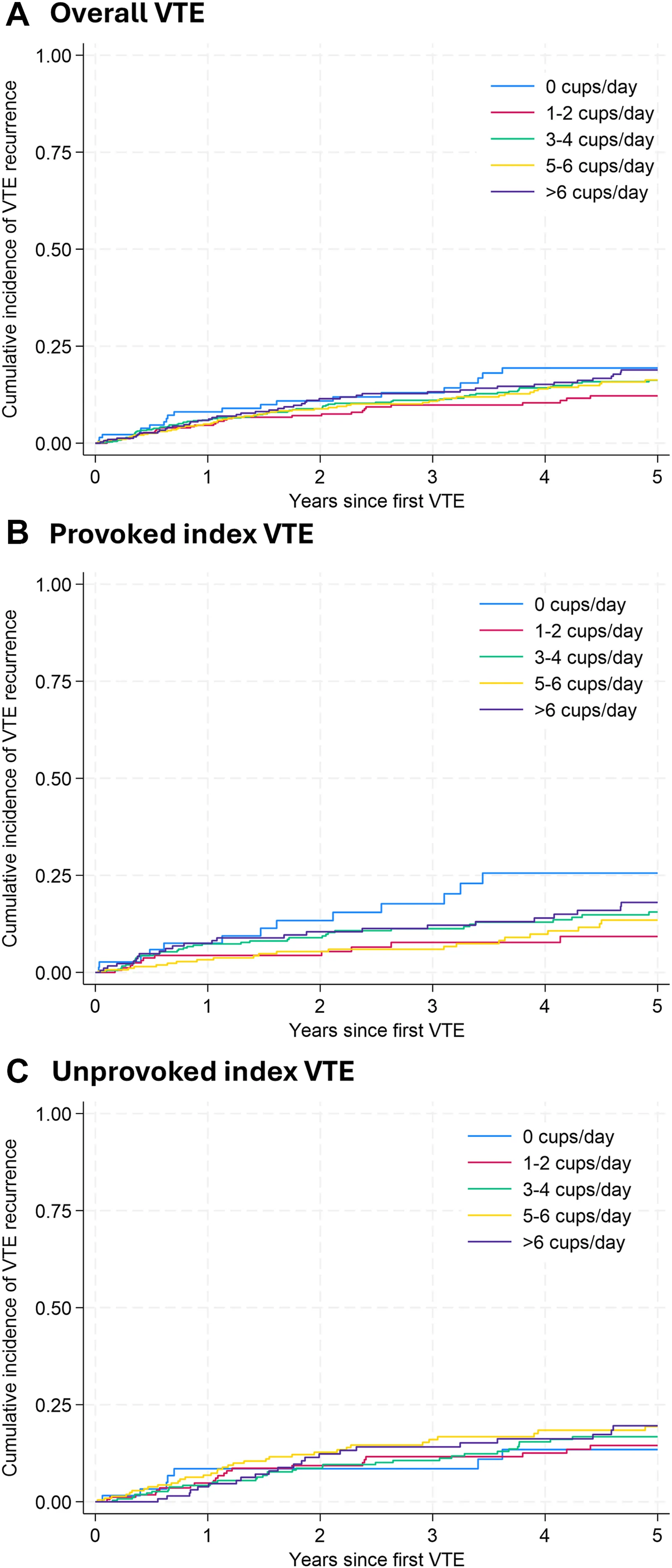
Cumulative incidence curves for overall venous thromboembolism (VTE) recurrence across categories of coffee consumption according to type of index VTE (overall, provoked, and unprovoked).

The IRs and HRs of recurrent VTE across categories of coffee consumption for overall VTE and stratified by the localization of index VTE (DVT and PE) are presented in Table 2. The recurrence risk was lowest among those consuming 1-2 cups of coffee per day (HR, 0.49; 95% CI, 0.30-0.81), whereas the HR was 0.74 (95% CI, 0.47-1.17) for those consuming >6 cups/d compared with noncoffee consumers in analyses adjusted for age and sex. Further adjustment for BMI, smoking and a history of CVD had negligible impact on the risk estimates. Stratified analysis for index DVT and PE showed a similar threshold pattern for the association between coffee consumption and risk of VTE recurrence.

**Table 2:** Incidence rates (IRs) and hazard ratios (HRs) of recurrent venous thromboembolism (VTE) according to subtypes (deep vein thrombosis [DVT] and pulmonary embolism [PE]) of the incident VTE event by categories of daily coffee consumption. The Trøndelag Health Study (HUNT) and Tromsø Study, 1994-2020.

Coffee consumption	Person-years	VTE recurrence	IR (95% CI)^a^	Model 1 HR (95% CI)^b^	Model 2 HR (95% CI)^c^
Index VTE					
0 cup/d	691	28	4.05 (2.80-5.86)	Ref.	Ref.
1-2 cups/d	1594	39	2.45 (1.79-3.35)	0.49 (0.30-0.81)	0.49 (0.30-0.81)
3-4 cups/d	2831	97	3.43 (2.81-4.18)	0.68 (0.44-1.06)	0.68 (0.44-1.04)
5-6 cups/d	2258	86	3.81 (3.08-4.71)	0.80 (0.52-1.23)	0.77 (0.50-1.20)
>6 cups/d	1969	60	3.05 (2.37-3.92)	0.74 (0.47-1.17)	0.71 (0.45-1.12)
Index DVT					
0 cup per day	335	15	4.48 (2.70-7.43)	Ref.	Ref.
1-2 cups/d	735	16	2.18 (1.33-3.56)	0.38 (0.18-0.79)	0.39 (0.19-0.80)
3-4 cups/d	1488	53	3.56 (2.72-4.66)	0.64 (0.35-1.16)	0.61 (0.34-1.12)
5-6 cups/d	1228	60	4.89 (3.79-6.29)	0.92 (0.51-1.66)	0.88 (0.49-1.58)
>6 cups/d	1161	39	3.36 (2.45-4.60)	0.74 (0.40-1.35)	0.66 (0.36-1.23)
Index PE					
0 cup/d	356	13	3.65 (2.12-6.28)	Ref.	Ref.
1-2 cups/d	860	23	2.68 (1.78-4.03)	0.62 (0.31-1.24)	0.60 (0.30-1.20)
3-4 cups/d	1343	44	3.28 (2.44-4.40)	0.73 (0.39.1.38)	0.72 (0.38-1.35)
5-6 cups/d	1030	26	2.53 (1.72-3.71)	0.58 (0.29-1.23)	0.59 (0.30-1.15)
>6 cups/d	808	21	2.60 (1.69-4.00)	0.71 (0.35-1.42)	0.75 (0.37-1.52)

Ref., Reference.

a IR, age-adjusted incidence rates per 100 person-years.

b HR, HRs adjusted for age and sex.

c HR, HRs adjusted for age, sex, body mass index (BMI), smoking, and cardiovascular disease (CVD).

The IRs and HRs for recurrent VTE across categories of coffee consumption were stratified by the type of index VTE events (unprovoked, provoked, and cancer-related VTE) and are displayed in Table 3. For index-unprovoked VTEs, we found no association between coffee consumption and VTE recurrence. For index-provoked VTEs, we found an inverse nonlinear association between coffee consumption and VTE recurrence. For provoked index VTE, the HR of recurrence was 0.33 (95% CI, 0.16-0.69) among those consuming 1 to 2 cups of coffee daily and 0.58 (95% CI, 0.47-1.17) for those consuming >6 cups/d, compared with noncoffee drinkers, after adjusting for age and sex. Further adjustment for BMI, smoking, and history of CVD had a minor impact on the risk estimates.

**Table 3:** Incidence rates (IRs) and hazard ratios (HRs) of recurrent venous thromboembolism (VTE) according to subtypes (unprovoked, provoked, and cancer-related VTE) of the incident VTE event by categories of daily coffee consumption. The HUNT and Tromsø studies, 1994-2020.

Coffee consumption	Person-years	VTE recurrence	IR (95% CI)^a^	Model 1 HR (95% CI)^b^	Model 2 HR (95% CI)^c^
Unprovoked index VTE					
0 cup/d	362	10	2.76 (1.48-5.13)	Ref.	Ref.
1-2 cups/d	932	25	2.68 (1.81-3.97)	0.75 (0.36-1.59)	0.72 (0.34-1.52)
3-4 cups/d	1359	48	3.53 (2.66-4.69)	0.98 (0.49-1.96)	0.96 (0.48-1.93)
5-6 cups/d	1109	48	4.33 (3.26-5.74)	1.29 (0.65-2.58)	1.26 (0.63-2.51)
> 6 cups/d	923	27	2.93 (2.01-4.27)	1.07 (0.52-2.21)	1.05 (0.50-2.20)
Provoked index VTE					
0 cup/d	329	18	5.47 (3.45-8.68)	Ref.	Ref.
1-2 cups/d	662	14	2.12 (1.25-3.57)	0.33 (0.16-0.69)	0.33 (0.16-0.69)
3-4 cups/d	1472	49	3.33 (2.52-4.41)	0.52 (0.30-0.92)	0.51 (0.29-0.91)
5-6 cups/d	1148	38	3.31 (2.41-4.55)	0.52 (0.29-0.93)	0.50 (0.28-0.89)
> 6 cups/d	1046	33	3.15 (2.24-4.44)	0.58 (0.32-1.03)	0.51 (0.28-0.93)

Ref., Reference.

a IR, age-adjusted incidence rates per 100 person-years.

b HR, HRs adjusted for age and sex.

c HR, HRs adjusted for age, sex, body mass index (BMI), smoking, and cardiovascular disease (CVD).

As the association between coffee consumption and VTE recurrence displayed a threshold pattern for all subgroup analyses, we conducted an analysis where coffee consumption was dichotomized into those who reported not to drink coffee and those who consumed ≥1 cup/d (Supplementary Figure and Table 4). The cumulative incidence curves for overall VTE recurrence for coffee consumers and noncoffee consumers across categories of first event VTE (overall, provoked, and unprovoked) displayed lower incidences for overall VTE recurrences, as shown in Table 4, where coffee consumers displayed lower risk of VTE recurrence compared with those who did not drink coffee across all subgroups of index VTE, except for unprovoked VTE. The lowered HRs for VTE recurrences by coffee consumption were particularly strong for provoked VTE events.

**Table 4:** Incidence rates (IRs) and hazard ratios (HRs) of recurrent venous thromboembolism (VTE) by daily coffee consumption (yes/no). The Trøndelag Health Study (HUNT) and Tromsø Study, 1994- 2020.

Coffee consumption	Person-years	VTE recurrence	IR (95% CI)^a^	Model 1 HR (95% CI)^b^	Model 2 HR (95% CI)^c^
Index VTE					
No coffee	691	28	4.05 (2.80-5.86)	Ref.	Ref.
Coffee	8652	282	3.26 (2.90-3.66)	0.70 (0.47-1.04)	0.68 (0.45-1.01)
Index DVT					
No coffee	335	15	4.48 (2.70-7.43)	Ref.	Ref.
Coffee	4611	168	3.64 (3.13-4.24)	0.71 (0.41-1.23)	0.67 (0.38-1-16)
Index PE					
No coffee	356	13	3.65 (2.12-6.28)	Ref.	Ref.
Coffee	4041	114	2.82 (2.35-3.39)	0.66 (0.37-1.19)	0.66 (0.37-1.19)
Unprovoked index VTE					
No coffee	362	10	2.76 (1.48-5.13)	Ref.	Ref.
Coffee	4324	148	3.42 (2.91-4.02)	1.04 (0.54-1.99)	1.01 (0.52-1.93)
Provoked index VTE					
No coffee	329	18	5.47 (3.45-8.68)	Ref.	Ref.
Coffee	4328	134	3.10 (2.61-3.67)	0.51 (0.31-0.85)	0.49 (0.29-0.81)

DVT, deep vein thrombosis; PE, pulmonary embolism; Ref., Reference.

a IR, age-adjusted incidence rates per 100 person-years.

b HR, HRs adjusted for age and sex.

c HR, HRs adjusted for age, sex, body mass index (BMI), smoking, and cardiovascular disease (CVD).

## 4 Discussion

In this study, we investigated the association between coffee consumption and the risk of recurrent VTE in a cohort of patients with VTE recruited from the general population. Overall, we found a nonlinear inverse association between coffee consumption and the risk of recurrent VTE. Consumption of 1 to 2 cups of coffee daily was associated with a 51% lower recurrence risk, while higher intake of coffee was associated with a 20% to 32% lower risk of recurrent VTE compared with noncoffee consumers in the analysis adjusted for age and sex. Further adjustment for BMI, smoking, and a previous history of CVD had minor impact on the risk estimates for recurrent VTE. The inverse association between coffee consumption and recurrent VTE was mainly driven by index-provoked VTEs, including cancer-related VTEs. Our findings suggest that coffee consumption may lower the risk of recurrent VTE, particularly for those with a provoked index event.

The association between coffee consumption and recurrent VTE has, to the best of our knowledge, previously not been investigated. Our findings are therefore unchallenged. However, our finding of a nonlinear association between coffee consumption and recurrence risk is in line with results from cohort- and case-control studies showing a nonlinear inverse association between coffee consumption and risk of incident VTE [[24], [25], [26]]. In a recent cohort study including 112,784 individuals recruited from the general population with repeated measurements of self-reported coffee consumption, coffee consumption displayed a nonlinear inverse association with VTE, where those drinking ≥1 cup of coffee per day had 19% to 23% lower risk of a first-lifetime VTE compared with noncoffee consumers [27]. In the present study, we demonstrated that the potential beneficial effect of coffee consumption on risk of incident VTE also applied to the risk of recurrent VTE, particularly for those with a provoked index event.

In the present study, we observed an inverse association between coffee consumption and recurrent VTE for provoked index VTE, but not for unprovoked index VTE. The observed discrepancy might be explained by the index event bias, which occurs because recurrence studies selectively include individuals who have already experienced an incident VTE [38]. Thus, the study population consists of individuals who possess several risk factors that contributed to the initial VTE event [39]. For individuals with persistent risk factors (eg, obesity or genetic predispositions like factor V Leiden), these risk factors remain in an individual following the incident VTE and continue to contribute to recurrence risk. In contrast, transient risk factors strongly associated with a provoked index VTE, such as immobilization or surgery, are often resolved after the incident event, thereby lowering the risk of recurrence [40]. As coffee consumption moderately lowered the risk of incident VTE, the antithrombotic properties of coffee appear to be sufficient to lower the risk of recurrence in individuals with a lower baseline risk of recurrence (eg, index-provoked VTE), but not in individuals with several persistent risk factors for VTE (eg, index-unprovoked VTE).

Results from cohort studies are influenced by potential misclassifications of the exposure (ie, coffee consumption), outcome (ie, VTE recurrence), and potential confounders due to the nonrandomized nature of these studies. In the present study, the categories of coffee consumption were extracted from self-administered questionnaires, which have been shown to have high validity [41]. However, lifestyle habits, such as coffee consumption, are highly modifiable and assumed to change over time. As we registered coffee consumption at different time intervals prior to the incident VTE event, it is likely that changes might have occurred in habits of coffee consumption until the time of the incident VTE. Such changes in coffee habits could lead to nondifferential misclassification of the exposure, with subsequent mitigation of the real association between coffee consumption and VTE recurrence [42]. Regarding ascertainment of VTE recurrence, we applied the same criteria for recurrent events as for incident VTE, including symptomatic and objectively confirmed events by radiology in need of restart of or prolonged anticoagulant treatment. In theory, different distributions of personal and VTE characteristics between noncoffee consumers and coffee consumers associated with recurrence risk might explain the observed inverse association between coffee consumption and VTE recurrence. However, the risk estimates for VTE recurrence were negligibly affected after multivariable adjustments for age, sex, BMI, smoking, and history of CVD. Nonetheless, due to the observational nature of our study, the possibility of residual confounding (eg, unmeasured and unrecognized confounders) cannot be excluded.

Previous studies have shown that coffee and constituents of coffee are implicated in several pathways in the pathogenesis of VTE. In the Multiple Environmental and Genetic Assessment of risk factors for venous thrombosis (MEGA) study [26], coffee consumers had lower plasma levels of coagulation factor (F)VIII and von Willebrand factor (VWF), both of which are strong and consistent risk factors for incident [43,44] and recurrent VTE [45,46]. Platelet reactivity is involved in the pathogenesis of incident VTE [47], and platelet inhibition reduces the risk of recurrent VTE [48]. Coffee and constituents of coffee, such as polyphenols, have been shown to inhibit platelet aggregation and platelet function ex vivo [[29], [30], [31]]. Further, the combination of high platelet reactivity and high plasma levels of VWF and FVIII is reported to have more than an additive effect on VTE risk [49,50]. Thus, it could be speculated that the inhibitory effect of coffee on both platelet reactivity and VWF/FVIII would have a synergistic beneficial impact on the risk of recurrent VTE.

This study has several strengths, including a large cohort of 2071 unselected patients with incident VTE derived from 2 population-based cohorts with high attendance rates, rigorous validation of incident and recurrent VTE events, and information on self-reported coffee consumption with high validity [41]. As coffee consumption was registered at various time intervals prior to the incident of VTE, it is likely that it did not accurately capture the daily intake of coffee at the VTE event. Such misclassification would presumably be nondifferential and lead to underestimation of the true association between coffee consumption and risk of recurrent VTE [42]. We lacked information on the duration of anticoagulant therapy after the incident of VTE events, which, if unevenly distributed between coffee abstainers and coffee consumers, might have impacted the recurrence risk. However, results remained similar when the start of follow-up was postponed up to 6 months after the incident VTE (sensitivity analysis). Furthermore, our study population consisted mainly of Caucasians, which limits generalizability to other ethnicities.

In conclusion, our study showed that intake of ≥1 cup of coffee daily was associated with lowered risk of recurrent overall VTE, particularly after provoked index VTE. As these findings are unchallenged, validation in other cohorts is warranted.
