# Supplementary material for: Association between coffee consumption and risk of recurrent venous thromboembolism – Results from the Trøndelag Health Study and Tromsø Study
Source: Res Pract Thromb Haemost. 2026 Sep 15;10(7):106966. doi: 10.1016/j.rpth.2026.106966 (PMC13657366; doi:10.1016/j.rpth.2026.106966)
Supplement: Supplementary file 1 [file mmc1.docx]

**Supplementary Material**

**Association between coffee consumption and risk of recurrent venous thromboembolism – Results from the HUNT and Tromsø studies**

Melliane M. Olsen^1,2^, Sigrid K. Brækkan^2,3^, Kristian Hveem^4-6^, Kjersti Grønning^1,7^, John-Bjarne Hansen^2,3^.

^1^Nord-Trøndelag Hospital Trust, Norway

^2^Thrombosis Research Group (TREC), Department of Clinical Medicine, UiT – The Arctic University of Norway, Tromsø, Norway

^3^Thrombosis Research Center (TREC), Division of Internal Medicine, University Hospital of North Norway, Tromsø, Norway

^4^HUNT Center for Molecular and Clinical Epidemiology, Department of Public Health and Nursing, Faculty of Medicine and Heath Science, Norwegian University of Science and Technology, Trondheim

^5^HUNT Research Center, Department of Public Health and Nursing, Norwegian University of Science and Technology, Levanger, Norway

^6^Department of Research, St. Olav University Hospital, Trondheim, Norway

^7^Department of Public Health and Nursing, Norwegian University of Science and Technology, Trondheim

**Supplementary Table 1.** Sensitivity analyses with follow-up starting 3- and 6 months after the index venous thromboembolism (VTE) date, respectively. Incidence rates (IRs) and hazard ratios (HRs) of recurrent VTE by categories of daily coffee consumption. The HUNT and Tromsø studies 1994-2020

|  | **Person-years** | | **VTE**  **recurrence** | **Crude**  **IR (95% CI)*** | **Model 1**  **HR (95% CI)**** | | **Model 2**  **HR (95% CI)***** | |
| --- | --- | --- | --- | --- | --- | --- | --- | --- |
| **Follow up starting 3 months after index VTE** | | | | | | | |  |
| 0 cup per day | | 659 | 25 | 3.79 (2.56-5.61) | Ref. | Ref. | |  |
| 1-2 cups per day | | 1 507 | 35 | 2.32 (1.68-3.24) | 0.50 (0.30-0.85) | 0.50 (0.29-0.85) | |  |
| 3-4 cups per day | | 2 668 | 89 | 3.34 (2.71-4.12) | 0.71 (0.45-1.13) | 0.70 (0.45-1.11) | |  |
| 5-6 cups per day | | 2 135 | 81 | 3.79 (3.05-4.72) | 0.85 (0.54-1.34) | 0.83 (0.52-1.31) | |  |
| > 6 cups per day | | 1 892 | 56 | 2.96 (2.28-3.85) | 0.77 (0.48-1.23) | 0.74 (0.46-1.21) | |  |
| **Follow up starting 6 months after index VTE** | | | | | | | |  |
| 0 cup per day | | 629 | 22 | 3.50 (2.30-5.31) | Ref. | Ref. | |  |
| 1-2 cups per day | | 1 427 | 30 | 2.10 (1.47-3.01) | 0.50 (0.28-0.87) | 0.49 (0.28-0.86) | |  |
| 3-4 cups per day | | 2 524 | 76 | 3.01 (2.41-3.77) | 0.70 (0.43-1.14) | 0.70 (0.43-1.14) | |  |
| 5-6 cups per day | | 2 025 | 74 | 3.65 (2.91-4.59) | 0.88 (0.54-1.43) | 0.86 (0.53-1.40) | |  |
| > 6 cups per day | | 1 820 | 52 | 2.86 (2.18-3.75) | 0.79 (0.48-1.31) | 0.77 (0.46-1.28) | |  |

IR: age-adjusted incidence rates per 100 person-years

HR*: hazard ratios adjusted for age and sex

HR***: hazard ratios adjusted for age, sex, BMI, smoking, CVD

Supplementary Fig.1 Cumulative incidence curves for overall VTE recurrence across categories of coffee consumption (coffee and no coffee) and first event VTE (overall, provoked, and unprovoked).
